# The effect of claudin-15 deletion on cationic selectivity and transport in paracellular pathways of the cecum and large intestine

**DOI:** 10.1038/s41598-023-33431-5

**Published:** 2023-04-26

**Authors:** Wendy Hempstock, Nozomi Nagata, Noriko Ishizuka, Hisayoshi Hayashi

**Affiliations:** 1 Laboratory of Physiology, Graduate School of Nutritional and Environmental Sciences, University of Shizuoka, 52-1 Yada, Suruga-ku, Shizuoka, Shizuoka 422-8526 Japan; 2 Department of Nursing, School of Nursing, University of Shizuoka, 52-1 Yada, Suruga-ku, Shizuoka, 422-8526 Japan

**Keywords:** Physiology, Gastroenterology

## Abstract

The large intestine plays a pivotal role in water and electrolyte balance. Paracellular transport may play a role in ion transport mechanisms in the cecum and large intestine; however, these molecular mechanisms and their physiological roles have not been fully studied. Claudin-15 forms a cation channel in tight junctions in the small intestine, but its role in the cecum and large intestine has not been investigated. This study aimed to explore the physiological role of claudin-15 in the cecum and large intestine using claudin-15 (Cldn15) KO mice. Electrical conductance, short-circuit current, Na^+^ flux, and dilution potential were assessed in isolated tissue preparations mounted in Ussing chambers. The induced short-circuit current of short-chain fatty acids, which are fermentative products in the intestinal tract, was also measured. Compared to wild type mice, the electrical conductance and paracellular Na^+^ flux was decreased in the cecum, but not the middle large intestine, while in both the cecum and the middle large intestine, paracellular Na^+^ permeability was decreased in Cldn15 KO mice. These results suggest that claudin-15 is responsible for Na^+^ permeability in the tight junctions of the cecum and large intestine and decreased Na^+^ permeability in the cecum may cause impaired absorption function.

## Introduction

The epithelial layer together with the junctions between the individual cells covers the inner surface of the gastrointestinal tract, separating the extracellular milieu from the internal compartments of the body and also acting as a barrier against noxious agents^1^. The small and large intestinal epithelia are thought to have similar functions in terms of barrier and electrolyte and water absorption^2^.

At tight junctions, which are the most luminal junctions between epithelial cells, the epithelial cells are “tightly” attached to each other. Classically, the properties of tight junctions in the epithelia have been assessed with electrophysiological techniques such as measuring electrical resistance in excised gastrointestinal preparations^1,3^. The permeability of the paracellular pathways of the small intestinal epithelium is high and so it is called a “leaky” epithelium. Compared to the small intestine, the electrical resistance of the tight junctions in the large intestine is relatively high, therefore it is classified as a “moderately tight” epithelia^1^.

Electrophysiological techniques can be used to distinguish “large” transcellular conductance, such as transport through K^+^ channels^4^, by using specific inhibitors in tight colonic epithelia. However, to date, there is no specific inhibitor of tight junctions, and as a result, the contribution of tight junctions to the overall electrical conductance of the epithelia has not been fully investigated in the large intestine^5^. Furthermore, it has been recently proposed that the “leaky paracellular pathway” acts as an energy saving mechanism to increase transcellular ion transport^6,7^. This idea suggests that paracellular Na^+^ transport in the cecum and proximal large intestine, which are “leaky” epithelium, may have a physiological role for ion transport.

It is also well known that the paracellular pathways of the small intestinal epithelium are cation-selective (P_Na_/P_Cl_ > 2.5–10)^1,3^. Recently, it was shown that the paracellular pathway is important for the glucose uptake process because it allows Na^+^ to be recycled back to the lumen for efficient nutrient uptake^8–10^. Similar to the small intestine, there are mechanisms of Na^+^-dependent nutrient absorption, including Na^+^-dependent short-chain fatty acid (SCFA) uptake in the cecum and large intestine^11–15^. On the other hand, little is known regarding the ion selectivity of paracellular pathways in the cecum and the large intestine. Early work by Frizzell and Schultz showed that the paracellular pathway is anion-selective in the rabbit distal large intestine (P_Na_/P_Cl_ = 0.6)^16^. Caco-2 cells, a cell line derived from human colorectal adenocarcinoma cells, have been reported to be cation-selective^17^ (see also table 25–1 in ref.^3^), and the proximal and distal large intestine in pigs^18^ and the proximal large intestine of mice^19^ were also reported to be cationic selective, but the molecular entity for this selectivity has not been identified.

Tight junctions (TJ) are complexes that are formed at the point where two or more cells meet. TJ are composed of strands of proteins including occludin and claudin family proteins, which determine the permselectivity of the tight junctions^20^. Claudin family proteins are a group of proteins possessing four transmembrane domains and two extracellular loops, of which 27 members have been discovered in mammals to date^21^, however many more may exist in nonmammalian species such as fish^22^. A number of claudin proteins have been classified according to their electrical properties in exogenous expression systems, such as cation channels (claudin-2, -10b, and -15), anion channels (claudin-10a), and barrier-forming (claudin-1, -3 and -5)^21,23^.

The importance of claudin-15 in the paracellular pathway in transepithelial transport has been reported previously^8–10,24^; however, focus was limited to the small intestine. Claudin-15 is expressed in the villi in the small intestine, and not in the crypts. Claudin-15 knockout (Cldn15 KO) mice have low luminal Na^+^ concentration, and lower transepithelial electrical conductance compared to wild type (WT) mice in the small intestine^10^, but the effect of claudin-15 deletion in the cecum and large intestine hasn’t been studied.

Claudin-15 is expressed in the crypts and not the surface cells in the cecum and large intestine^25,26^. So, it raises the question, what is the reason for this difference in expression pattern in the cecum and large intestine compared to the small intestine? Since the role of the paracellular pathway in the cecum and large intestine has not been fully clarified, and the ion selectivity of the tight junctions in the cecum and large intestine haven’t been thoroughly studied, the goal of this research was to examine the role of the claudin-15 in the paracellular pathway of the cecum and large intestine using claudin-15 knockout (Cldn15 KO) mice.

## Results

### Tissue localization of claudin-15 in the cecum and large intestine

**Figure 1:**
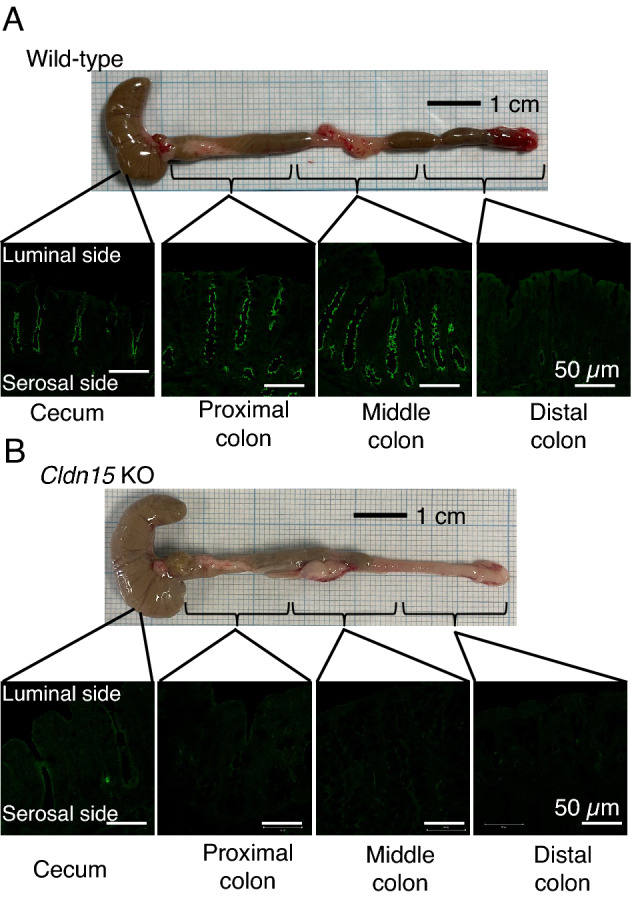
Claudin-15 expression in the cecum and large intestine. Representative immunofluorescence images showing claudin-15 (green) antibody staining in the cecum, proximal, middle, and distal large intestine of WT mice (**A**) and Cldn15 KO mice (**B**). Scale bar, 50 µm.

The segmental difference of claudin-15 in the large intestine has not been determined, so we examined the tissue distribution of claudin-15 protein in the cecum and each section of the large intestine by immunofluorescence (Fig. 1A). As shown in Fig. 1A, claudin-15 is exclusively expressed in the crypts but not surface cells from the cecum to the distal large intestine, and the expression intensity decreases gradually from the cecum to the distal segment of the large intestine.

### The luminal environment is perturbed in the cecum and large intestine of claudin-15 knockout mice

**Figure 2:**
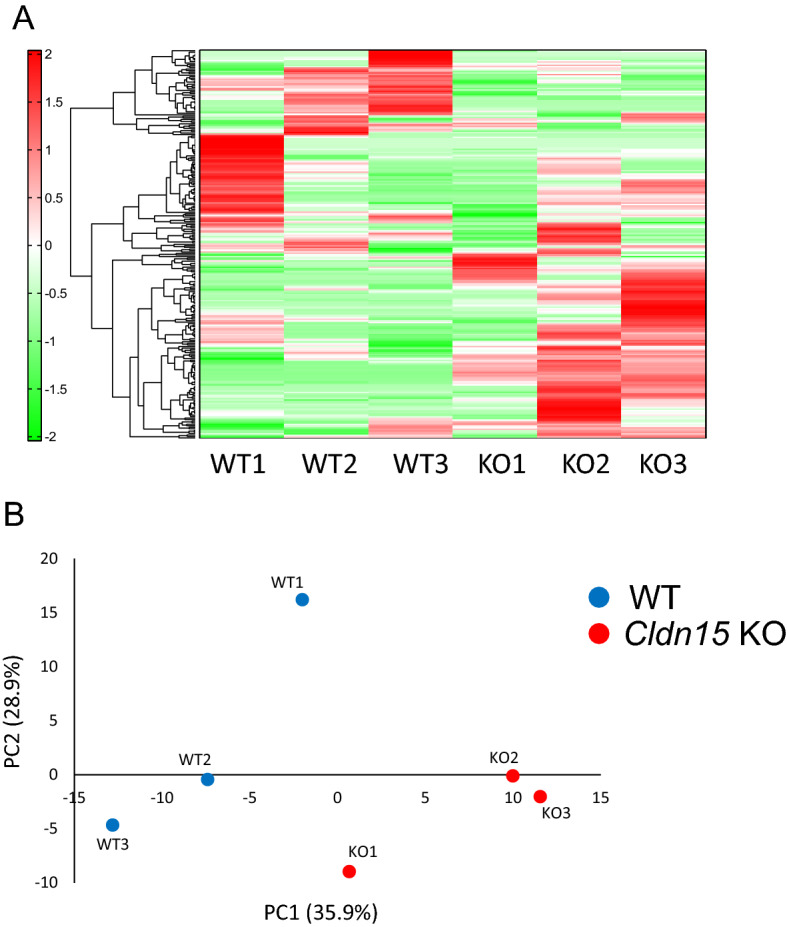
Metabolomic analysis of cecal contents. The contents of the cecum were collected from Cldn15 KO and WT mice. The contents were weighed and then the water-soluble metabolites were extracted and analyzed by CE-TOFMS. (**A**) Hierarchical clustering heat map showing the patterns of intestinal metabolite between the samples. Green and red show lower and higher concentrations respectively. Heatmap was created by Human Metabolome Technologies, Inc. (HMT) using proprietary software developed in-house. (**B**) Principal component analysis of the metabolome data from the cecal luminal contents from Cldn15 KO (red dots) and WT (blue dots) mice.

The cecum of claudin-15 knockout (Cldn15 KO) mice is enlarged (Fig. 1B) and the amount of luminal content was increased 2.7 times compared to WT mice, which has been published previously by Tamura et al.^27^. This result indicates that malabsorption may be occurring in the small intestine of Cldn15 KO mice. To assess whether nutrient malabsorption is occurring, the cecal luminal contents of Cldn15 KO and WT mice were collected and analyzed by CE-TOF (capillary electrophoresis time-of-flight) mass spectroscopy. As shown in heat map and principal component analysis (PCA), cecal luminal contents differ between Cldn15 KO and WT mice (Fig. 2A,B). However, the data did not support the idea that Cldn15 KO mice have malabsorption (Tables 1, 2). The distinct metabolic profiles of the luminal contents (Fig. 2A) suggest that the loss of claudin-15 alters the luminal environment by causing low luminal Na^+^ levels and as a result, the microbiota population may change as well.

**Table 1:** Compounds found to be higher in the cecal luminal contents of Cldn15 KO mice.

Compound	WT	Cldn15 KO	Ratio (KO vs. WT)	P
1-methyl-4-imidazoleacetic acid	4.2 × 10^–4^ (1.3 × 10^–4^)	1.2 × 10^–3^ (9.4 × 10^–5^)*	2.8	0.00202
*N*-methylproline	5.1 × 10^–4^ (1.2 × 10^–4^)	4.7 × 10^–3^ (9.2 × 10^–4^)*	9.4	0.0141
Pipecolic acid	4.3 × 10^–4^ (1.6 × 10^–4^)	1.1 × 10^–3^ (2.1 × 10^–4^)*	2.5	0.0173
2-hydroxyglutaric acid	3.5 × 10^–4^ (2.5 × 10^−4^)	1.0 × 10^–3^ (2.2 × 10^–4^)*	2.9	0.0256
Malic acid	2.7 × 10^–4^ (7.2 × 10^–5^)	9.2 × 10^–4^ (2.4 × 10^–4^)*	3.4	0.0353
Cytidine	2.2 × 10^–4^ (1.4 × 10^–5^)	5.1 × 10^–4^ (1.0 × 10^–4^)*	2.4	0.0367
Azelaic acid	1.2 × 10^–4^ (3.3 × 10^–5^)	2.2 × 10^–4^ (4.5 × 10^–5^)*	1.8	0.0401
β-alanine	5.6 × 10^–4^ (8.0 × 10^–5^)	1.1 × 10^–3^ (2.4 × 10^–4^)*	1.9	0.0491
Piperdine	2.8 × 10^–5^ (1.1 × 10^–5^)	5.2 × 10^–5^ (3.6 × 10^–6^)	1.8	0.0529
Trimethylamine	2.5 × 10^–3^ (1.5 × 10^–3^)	1.0 × 10^–2^ (3.7 × 10^–3^)	4.0	0.0566
Glucose-1-phosphate	6.7 × 10^–5^ (4.8 × 10^–5^)	2.1 × 10^–4^ (7.5 × 10^–5^)	3.2	0.0588
Guanosine	1.3 × 10^–4^ (3.9 × 10^–5^)	3.1 × 10^–4^ (9.6 × 10^–5^)	2.4	0.0672
Uric acid	3.9 × 10^–5^ (2.4 × 10^–5^)	1.0 × 10^–4^ (3.7 × 10^–5^)	2.7	0.0735
Butyric Acid	3.3 × 10^–2^ (6.9 × 10^–3^)	4.5 × 10^–2^ (2.9 × 10^–3^)	1.4	0.0796
2’-Deoxycytidine	2.4 × 10^–5^ (4.8 × 10^–6^)	4.3 × 10^–5^ (1.1 × 10^–5^)	1.8	0.0837
5-hydroxypentanoic acid	5.0 × 10^–5^ (8.1 × 10^–6^)	6.6 × 10^–5^ (1.0 × 10^–5^)	1.3	0.0992
Anserine	3.6 × 10^–5^ (2.6 × 10^–6^)	9.3 × 10^–4^ (5.4 × 10^–4^)	26	0.103
Glutaric acid	2.4 × 10^–4^ (6.4 × 10^–5^)	7.1 × 10^–4^ (3.0 × 10^–4^)	3.0	0.106

Data are expressed as the mean (SD) relative area of the peak for each metabolite for WT (n = 3) and Cldn15 KO (n = 3). The relative ratio compares the mean KO value vs the mean WT value. Welch’s *T*-test was used to compare the means, P < 0.05 was considered to be significantly different.

**Table 2:** Compounds found to be lower in the cecal luminal contents of Cldn15 KO mice.

Compound	WT	Cldn15 KO	Ratio (KO vs. WT)	P
Cytidine monophosphate	8.1 × 10^–5^ (1.0 × 10^–5^)	2.3 × 10^–5^ (1.3 × 10^–6^)*	0.3	0.00865
5-hydroxyindoleacetic acid	1.0 × 10^–3^ (1.9 × 10^–4^)	4.0 × 10^–4^ (1.4 × 10^–4^)*	0.4	0.0130
Spermidine	1.1 × 10^–3^ (1.9 × 10^–4^)	5.5 × 10^–4^ (1.4 × 10^–4^)*	0.5	0.0135
Asymmetric dimethylarginine	2.5 × 10^–4^ (5.9 × 10^–5^)	1.1 × 10^–4^ (1.6 × 10^–5^)*	0.4	0.0476
Nicotinamide ribotide	3.5 × 10^–4^ (6.8 × 10^–5^)	1.3 × 10^–4^ (1.1 × 10^–4^)	0.4	0.0503
*N*^1^, *N*^8^-Diacetylspermidine	9.3 × 10^–5^ (2.5 × 10^–5^)	4.3 × 10^–5^ (1.3 × 10^–5^)	0.5	0.0512
4-pyridoxic acid	2.3 × 10^–4^ (3.7 × 10^–5^)	1.6 × 10^–4^ (3.3 × 10^–5^)	0.7	0.0643
Adenosine monophosphate	1.8 × 10^–4^ (7.9 × 10^–5^)	3.2 × 10^–5^ (1.5 × 10^–5^)	0.2	0.0767
Trimethyllysine	1.2 × 10^–4^ (1.4 × 10^–5^)	5.4 × 10^–5^ (3.9 × 10^–5^)	0.4	0.0813
3-(4-Hydroxyphenyl)propionic acid	2.4 × 10^–3^ (9.0 × 10^–4^)	8.8 × 10^–4^ (1.7 × 10^–4^)	0.4	0.0957
Glutamine	1.4 × 10^–3^ (4.3 × 10^–4^)	7.2 × 10^–4^ (4.0 × 10^–4^)	0.5	0.105
5-thymidylic acid	4.8 × 10^–5^ (1.6 × 10^–5^)	2.2 × 10^–5^ (3.0 × 10^–6^)	0.5	0.108

Data are expressed as the mean (SD) relative area of the peak for each metabolite for WT (n = 3) and Cldn15 KO (n = 3). The relative ratio compares the mean KO value vs the mean WT value. Welch’s T-test was used to compare the means, P < 0.05 was considered to be significantly different.

**Figure 3:**
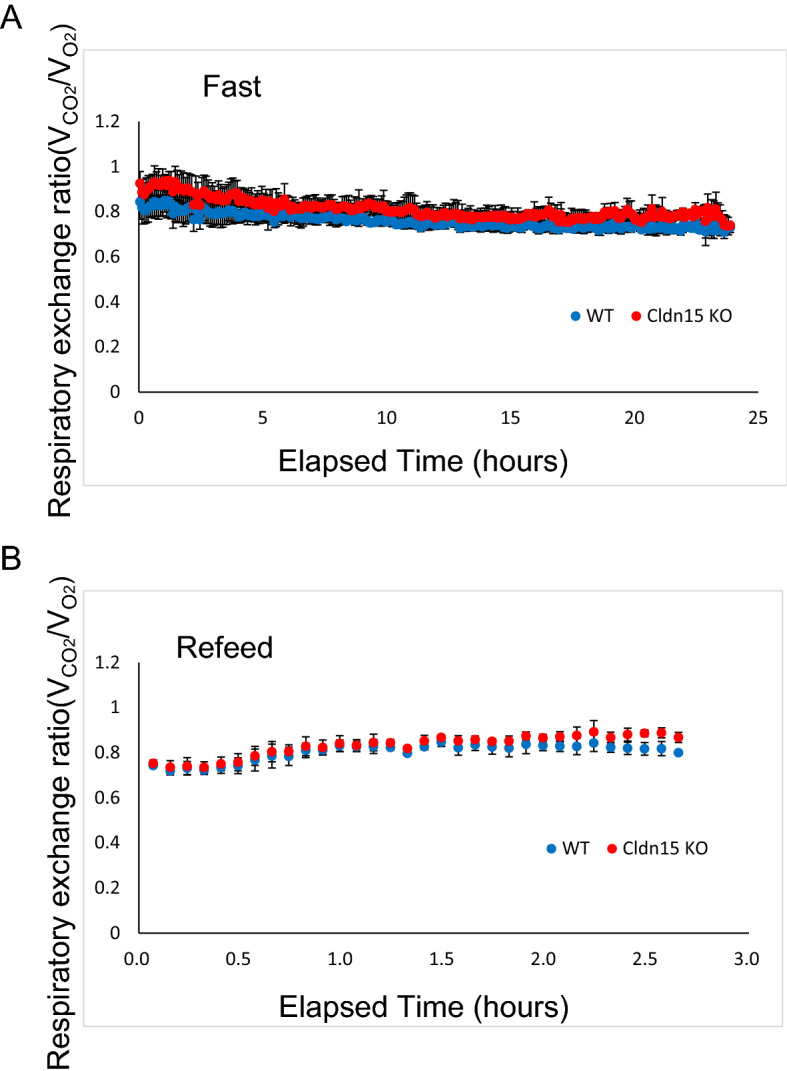
Energy metabolism in claudin-15 knockout and WT mice. Cldn15 KO (n = 3) and WT (n = 3) mice were placed into metabolic cages and after a period of adjustment, the resting quotient (RQ) was measured by indirect calorimetry for (**A**) fasting and (**B**) re-feeding. Data are expressed as the mean for each time point and the error bars are the SD.

To investigate the metabolic consequences of the deletion of claudin-15, Cldn15 KO mice were subjected to indirect calorimetry. Oxygen consumption and carbon dioxide production were measured to determine the respiratory quotient (RQ) which reflects carbohydrate and fat oxidation. Mice were fasted for 24 h and were refed at 9:00 on the day of the experiment. Fasting resulted in an RQ of about 0.7 (Fig. 3A) indicating fat metabolism in both WT and Cldn15 KO mice. After re-feeding, the RQ became about 1 (Fig. 3B), indicating the occurrence of carbohydrate metabolism. No significant change in the time required for adaptation to carbohydrate metabolism was observed between WT and Cldn15 KO mice. In addition, no significant differences in recorded locomotion activity were observed between WT and Cldn15 KO mice. These data, accompanied by the outwardly normal maturation and development of these mice and the lack of major nutritional impairment^27^, suggest that the adaptive hypertrophy of the small intestine in Cldn15 KO mice allows for adequate digestion and absorption of nutrients.

**Figure 4:**
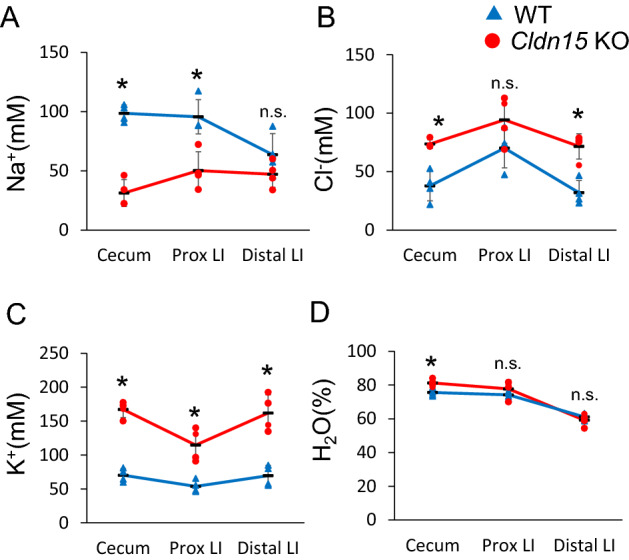
Electrolyte and water percentage in the luminal contents. The contents of each luminal segment were collected from Cldn15 KO mice (n = 4) and WT (n = 4), and the H_2_O content and ion concentrations were measured. (**A**) Na^+^ concentration; (**B**) Cl^−^ concentration; (**C**) K^+^ concentration; (**D**) H_2_O content. The dots represent the individual points and the black bars represent the mean, error bars represent the SD. Statistical significance from left to right: (**A**) *P = 0.029; *P = 0.029; n.s. P = 0.200; (**B**) *P = 0.029; n.s. P = 0.343; *P = 0.029; (**C**) *P = 0.029; *P = 0.029; *P = 0.029; (**D**) *P = 0.029; n.s. P = 0.343; n.s. P = 0.686, Mann–Whitney *U* test, Cldn15 KO vs. WT. *Prox LI* proximal large intestine, *Distal LI* distal large intestine, Please see supplementary table 1 for data points.

The ionic composition of the intestinal luminal contents is the result of differences between intestinal fluid secretion and absorption mechanisms. Transepithelial electrolyte and water transport depend on cellular mechanisms and paracellular shunt pathways. To gain insight into the electrolyte and water transport of each segment, we measured ion concentrations of luminal contents from the cecum to the distal large intestine (Fig. 4, Supplementary Table 1). The Na^+^ concentration of luminal contents gradually decreased from the cecum to the distal colon in WT mice (Fig. 4A). Luminal Na^+^ concentration was decreased in the cecum (p = 0.029) and proximal large intestine (p = 0.029) of Cldn15 KO mice compared to WT mice, but there was no difference in the distal large intestine (p = 0.200). This suggests that epithelial Na^+^ transport mechanisms are attenuated in the cecum and large intestine of Cldn15 KO mice. The concentration of Cl^−^ in the luminal contents increased markedly from the cecum to distal large intestine in Cldn15 KO mice (Fig. 4B, p = 0.029, 0.343, 0.029, cecum, proximal, and distal large intestine, respectively), suggesting epithelial Cl^−^ transport mechanisms in the cecum and large intestine are augmented. The K^+^ concentration (Fig. 4C) was increased in Cldn15 KO mice (p = 0.029, 0.029, 0.029 in the cecum, proximal, and distal large intestine, respectively). This result implies that K^+^ (re)absorption in the cecum and large intestine may be decreased in Cldn15 KO mice. The water content in the cecum of Cldn15 KO mice was higher than that of WT mice (Fig. 4D, p = 0.029). However, this difference disappeared in the large intestine, suggesting water absorption may be upregulated in the large intestine of Cldn15 KO mice.

### Transepithelial electrical conductance is decreased in the cecum of claudin-15 knockout mice

**Figure 5:**
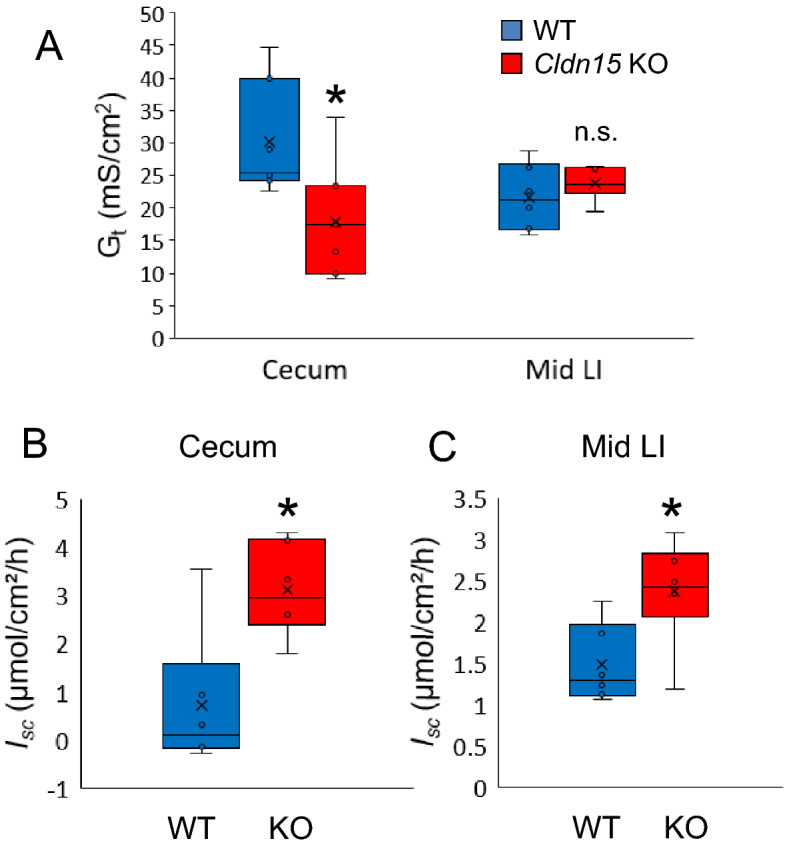
Baseline electrical parameters in the cecum and middle large intestine. (**A**) The baseline transmembrane electrical conductance (*G*_*t*_) of the cecum and middle large intestine was measured under short-circuit conditions in Ussing chambers (n = 6 (including 1 female) and 7, KO and WT, respectively). The baseline short-circuit current (*I*_*sc*_) was measured in the (**B**) cecum and (**C**) the middle large intestine. The box plots represent the minimum, first quartile, median, third quartile, and maximum values, the mean is marked with an X, n = 5 (including 1 female) and 6 for KO and WT, respectively for figures (**B**) and (**C**). Statistical significance from left to right: (**A**) *P = 0.017; n.s. P = 0.485; (**B**) *P = 0.026; (**C**) *P = 0.026, Mann–Whitney *U* test, Cldn15 KO vs. WT. Please see supplementary table 2 for data points.

The basic electrical parameters of the cecum and large intestine were investigated by Ussing chambers using Cldn15 KO mice and WT mice as controls (Fig. 5, Supplementary Table 2). Transepithelial electrical conductance (*G*_*t*_), which is an indicator of epithelial leakiness, was decreased in the cecum (p = 0.017) but not in the middle large intestine (Fig. 5A). In contrast, baseline short-circuit current (*I*_*sc*_), was increased in both the cecum (p = 0.026) and middle large intestine (p = 0.026) of Cldn15 KO mice (Fig. 5B,C). These results suggest that a loss of paracellular pathways (as indicated by the lower *G*_*t*_) in Cldn15 KO mice may be compensated by transcellular transport (indicated by the increase in *I*_*sc*_).

### Paracellular and transcellular Na^+^ flux is decreased in the cecum of claudin-15 knockout mice

**Figure 6:**
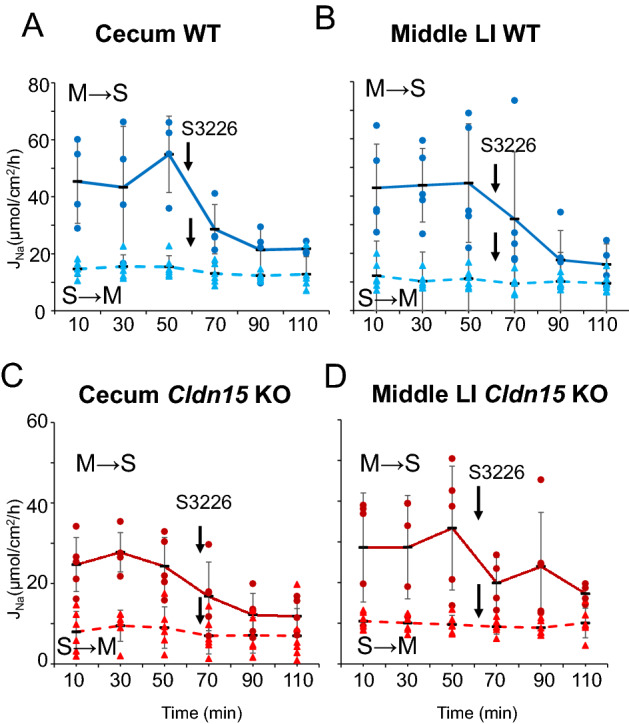
Measurement of unidirectional flux of Na^+^ in the cecum and middle large intestine. Intestinal sheets for the cecum and middle large intestine were prepared by removing the muscle layer and then they were mounted in Ussing chambers. The M→S and S→M unidirectional flux of Na^+^ was measured for each segment. (**A**) Na^+^ flux after each 20-min period in the WT cecum and (**B**) middle large intestine of WT mice. (**C**) Cecum of Cldn15 KO mice. (**D**) middle large intestine of Cldn15 KO mice. The dots represent the individual points and the black bars represent the mean, n = 3–6 (KO: including 1 female). Error bars represent the SD. Please see supplementary table 3 for data points.

The above-mentioned lower luminal Na^+^ concentration and transepithelial *G*_*t*_ decrease in Cldn15 KO mice suggested that there is a decrease of transepithelial Na^+^ pathways. Transcellular Na^+^ absorption involves electro-neutral Na^+^/H^+^ exchange that mainly occurs via electroneutral Na^+^/H^+^-exchange transporter isoform 3 (NHE3)^28^.To investigate transepithelial Na^+^ transport in detail in the cecum and middle large intestine, the unidirectional fluxes of Na^+^ in both the mucosal to serosal (M→S) and serosal to mucosal (S→M) directions were assessed by Ussing chambers. Na^+^ flux was measured over three 20-min periods, followed by luminal addition of the NHE3 specific inhibitor S3226^29^ and 3 subsequent 20-min periods (Fig. 6, Supplementary Tables 3 and 6). In the cecum of WT mice (Fig. 6A, Table 3), basal unidirectional M→S flux (47.9 (13.0) µmol/cm^2^/h) was larger than that of S→M flux (15.2 (3.5) µmol/cm^2^/h). In addition, the basal *I*_*sc*_ was much smaller than these values of flux (Fig. 5B, 0.71 µmol/cm^2^/h), suggesting that the electroneutral Na^+^ absorption process is a major Na^+^ absorption system in the cecum (> 30 µmol/cm^2^/h), corresponding to previous studies^30–32^. Upon the addition of S3226, only M→S flux was profoundly attenuated (Fig. 6, Table 3). These results suggest that Na^+^ is absorbed mainly via NHE3 in the cecum. In the cecum of Cldn15 KO mice (Fig. 6C, Table 3), both basal S→M (p = 0.016) and M→S (p = 0.015) fluxes were decreased compared to WT mice. In addition, the magnitude of inhibition by S3226 was higher in WT mice in M→S flux (S3226-sensitive component, 11.9 (7.4) µmol/cm^2^/h and 23.0 (9.0) µmol/cm^2^/h, KO and WT, p = 0.190). These results suggest that both paracellular Na^+^ pathways and NHE3-mediated Na^+^ absorption mechanisms are decreased in the ceca of Cldn15 KO mice.

**Table 3:** Unidirectional Na^+^ flux before and after addition of S3226.

Segment	Na^+^ flux Direction	WT (n = 4–6) µmol/cm^2^/h ± SD	Cldn15 KO (n = 5–6) µmol/cm^2^/h ± SD	P (Mann–Whitney *U* test)
Cecum	M → S	47.9 ± 13.0	25.6 ± 5.9*	0.016
	S → M	15.2 ± 3.5	8.8 ± 4.2*	0.015
Middle LI	M → S	43.7 ± 16.1	30.2 ± 13.5	0.548
	S → M	11.2 ± 3.4	10.1 ± 1.9	0.818
Cecum (post-S3226)	M → S	24.8 ± 6.4	13.6 ± 5.8*	0.032
	S → M	12.3 ± 3.1	7.0 ± 5.1	0.065
Middle LI (post-S3226)	M → S	26.5 ± 18.6	16.9 ± 5.1	0.421
	S → M	9.7 ± 2.7	9.4 ± 2.2	1.000

Intestinal sheets for the cecum and middle large intestine were prepared by removing the muscle layer and then they were mounted in Ussing chambers. The M→S and S→M unidirectional flux of Na^+^ was measured for each segment. The values in the table represent the average flux ± SD for each segment before and after the addition of the NHE3 inhibitor S3226. The values represent the mean, n = 4–6 for WT and 5–6 for Cldn15 KO (including 1 female). Please see Supplementary Table 6 for data points.

In the middle large intestine of WT mice, basal unidirectional M→S flux was larger than that of S→M flux (Fig. 6B, Table 3). The basal *I*_*sc*_ was smaller than those flux values (Fig. 5C, 1.5 (0.5) µmol/cm^2^/h), indicating electroneutral Na^+^ absorption is a major Na^+^ absorption mechanism in the middle large intestine, as in the cecum. In accordance with these results, the addition of S3226 to the luminal side resulted in a decrease of M→S flux (Fig. 6B, Table 3). In Cldn15 KO mice, M→S Na^+^ flux did decrease after the addition of S3226 (Fig. 6D, Table 3); however the magnitude of net inhibition was not different compared to WT mice (S3226-sensitive component, 13.3 (11.7) µmol/cm^2^/h and 17.3 (14.5) µmol/cm^2^/h, KO and WT, p = 0.841). In the middle large intestine of Cldn15 KO mice, basal S→M flux was not decreased as compared with WT mice (p = 0.818) (Fig. 6D, Table 3), suggesting that the paracellular Na^+^ pathway was not decreased in middle large intestine of Cldn15 KO mice.

### The paracellular Na^+^ selectivity is decreased in the cecum and large intestine of claudin-15 knockout mice

**Figure 7:**
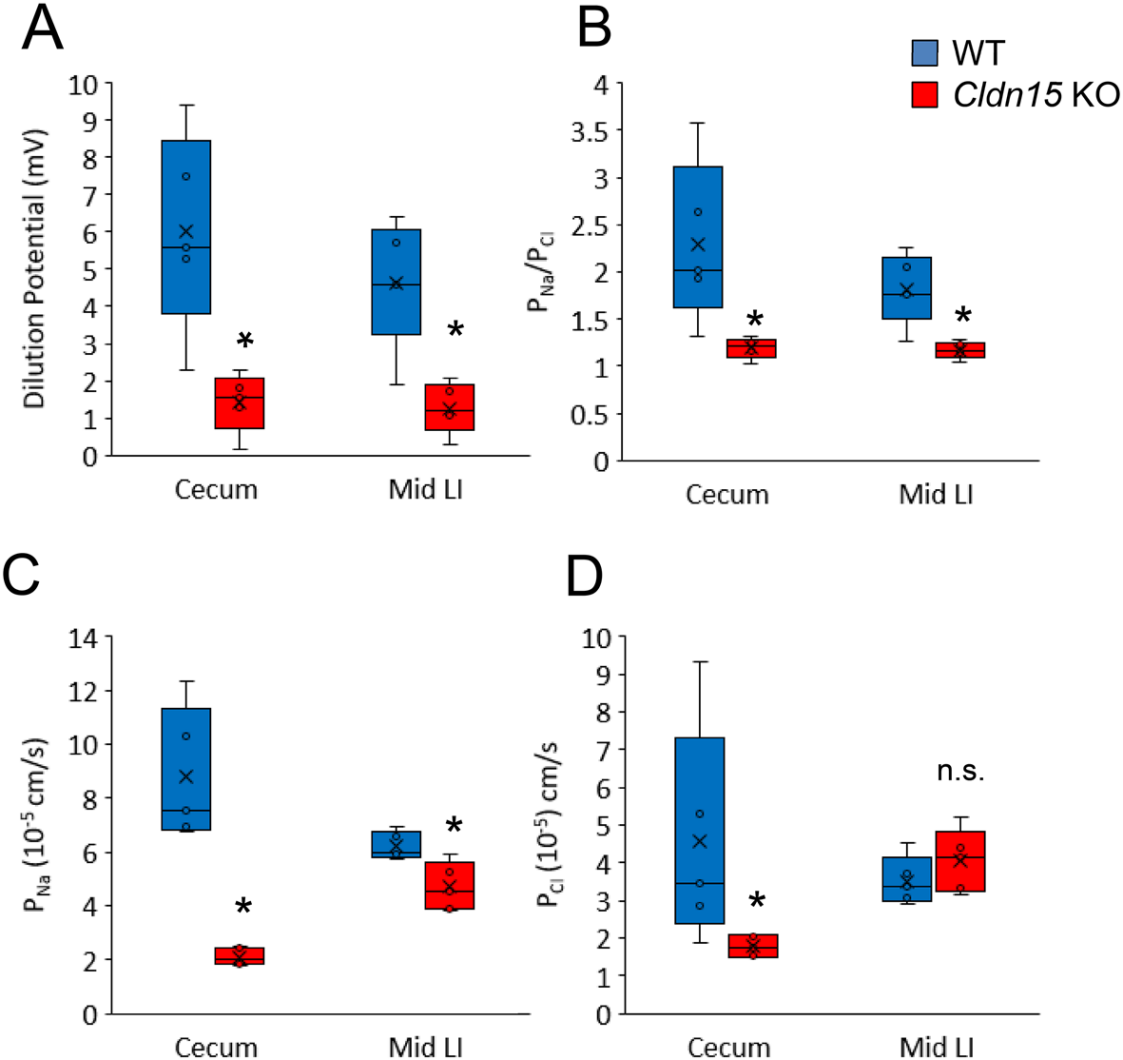
The relative permeability of Na^+^ is reduced in the cecum and proximal and middle large intestine of claudin-15 KO mice. The cecum and middle large intestine were prepared and mounted in Ussing chambers. (**A**) Transmural potential difference (∆mV) after dilution of NaCl on the mucosal side in the cecum and middle large intestine. (**B**) The relative permeability of Na^+^ in the cecum and middle large intestine. (**C**) The permeability of Na^+^ (P_Na_) and (**D**) Cl^−^ (P_Cl_) in the cecum and middle large intestine of Cldn15 KO mice. The box plots represent the minimum, first quartile, median, third quartile, and maximum values, the mean is marked with an X, n = 5. Statistical significance from left to right: (**A**) *P = 0.008; *P = 0.016; (**B**) *P = 0.016; *P = 0.016; (**C**) *P = 0.008; *P = 0.032; (**D**) *P = 0.032; n.s. P = 0.421, Mann–Whitney *U* test, Cldn15 KO vs. WT. Please see supplementary table 4 for data points.

To characterize permselectivity in the cecum and large intestine, the dilution potential of NaCl was measured (Fig. 7, Supplementary Table 4). It is thought that the TJ generally have cationic selectivity in leaky epithelia such as the small intestine and gallbladder^33^, however the large intestinal epithelia is tighter and the permselectivity has not been fully investigated. Since Na^+^ is efficiently absorbed against a steep gradient by epithelial sodium channel (ENaC) in the distal colon, the lumen negative potential is thought to be dissipated via paracellular Cl^−^ diffusion^33^, implying the permselectivity may be anionic. We measured the relative permeabilities of Na^+^ (P_Na_) or Cl^−^ (P_Cl_) and the dilution potential of isolated segments of the cecum and large intestine using Ussing chambers. Dilution of the M (mucosal) side of the chamber in open circuit conditions, results in the influx of NaCl from the S (serosal) side, which results in change of the membrane potential difference (PD)^34,35^.

Upon dilution of the M side, the PD decreased in the cecum (p = 0.008) and the middle large intestine (p = 0.016) compared to WT controls (Fig. 7A). Relative permeability of Na^+^ (P_Na_/P_Cl_) was calculated by Goldman-Hodgkin-Katz equation using dilution PD, and it was decreased in the cecum (p = 0.016) and in the middle large intestine (p = 0.016) (Fig. 7B). The absolute permeability coefficients of Na^+^ (P_Na_) and Cl^−^ (P_Cl_) were calculated with the Kimizuka-Koketsu equation^36^. In the cecum (p = 0.008) and middle large intestine (p = 0.032) P_Na_ was decreased (Fig. 7C), while P_Cl_ was only decreased in the cecum (p = 0.032, Fig. 7D). From these results, it can be reasoned that the loss of claudin-15 leads to decreased paracellular permeability of Na^+^, and the results also show that in WT mice, the permselectivity favors the passage of Na^+^ over Cl^−^ in the cecum and large intestine.

### The cellular localization of claudin-2 and claudin-7 in the cecum and large intestine

**Figure 8:**
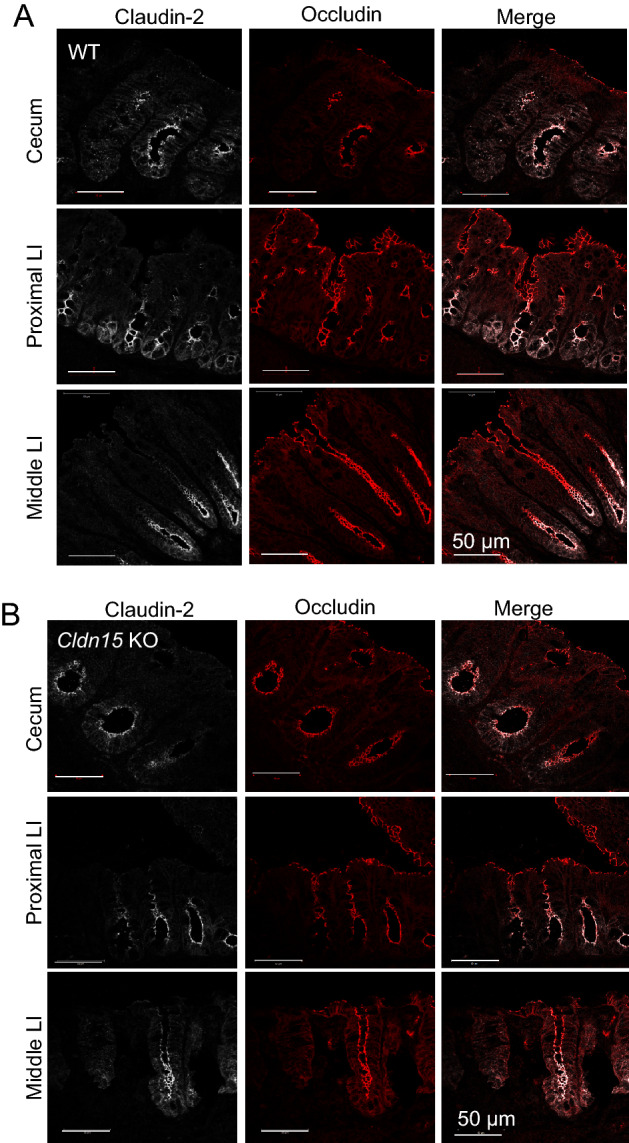
Immunofluorescence of claudin-2 and occludin in the cecum and large intestine. Representative immunofluorescence images showing claudin-2 (white) and occludin (red) antibody staining in the cecum, proximal and middle large intestine for (**A**) WT mice and (**B**) Cldn15 KO mice. Scale bar, 50 µm.

**Figure 9:**
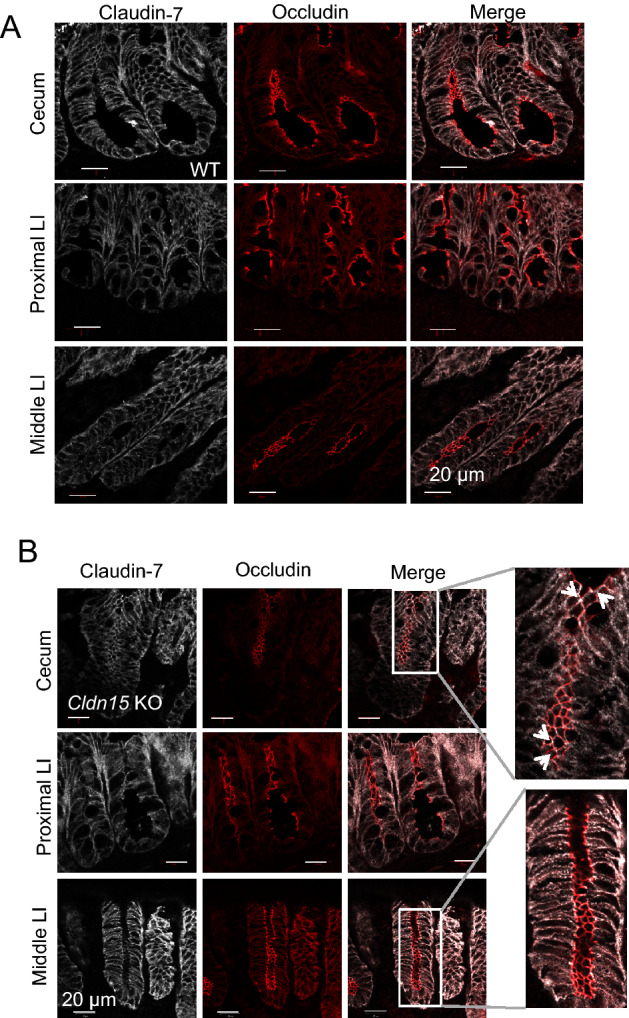
Immunofluorescence localization of claudin-7 in the cecum and large intestine. Representative immunofluorescence images showing claudin-7 (white) and occludin (red) antibody staining in the cecum, proximal and middle large intestine for (**A**) WT mice and (**B**) Cldn15 KO mice. Scale bar, 20 µm. Arrowheads point to localization of claudin-7 to the tight junctions.

Since the paracellular selectivity of Na^+^ was decreased in the cecum and large intestine of Cldn15 KO mice (Fig. 7), but the *G*_*t*_ and S→M Na^+^ flux were decreased only in the cecum (Figs. 5A and 6, Table 3), the effect of the loss of claudin-15 seems to be dependent on the intestinal segment. To investigate the reason for this difference, the expression of claudin-2 and claudin-7 in the cecum and large intestine was investigated using immunofluorescence. Claudin-2 is expressed in the large intestine TJ and has been found to have cationic selectivity^34,37,38^. Claudin-7 is also one of the predominant claudins in the large intestine and it has been shown that deletion of claudin-7 enhances the paracellular permeability in the colon^39^. In WT mice, claudin-2 is expressed in the crypts of the cecum and the large intestine (Fig. 8A), while claudin-7 is expressed diffusely throughout the cell and in the basolateral membrane rather than being specifically localized to the TJ (Fig. 9A). In Cldn15 KO mice, the localization of claudin-2 did not seem to change (Fig. 8B), while claudin-7 appeared to localize more to the tricellular TJ only in the cecum of Cldn15 KO mice (Fig. 9B, white arrows in inset). Together, the loss of claudin-15 in the cecum leads to decreased Na^+^ flux, conductance, and permeability of Na^+^. However, the compensation by claudin-7 suggests that claudin-15 may play an important role in the cecum.

### Short chain fatty acid-induced short-circuit current in the cecum and large intestine of claudin-15 knockout mice

**Figure 10:**
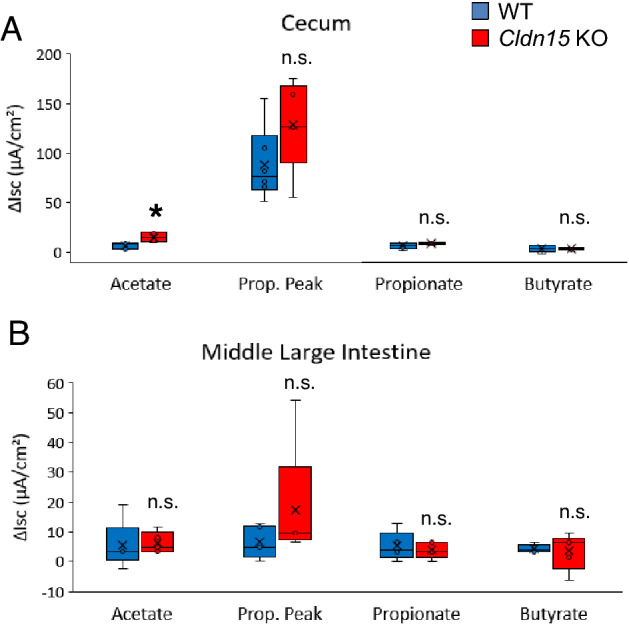
Short chain fatty acid-induced short-circuit current in the cecum and large intestine. Short-circuit current increases were measured in Ussing chambers after addition of 10 mM of acetate, propionate, or butyrate to the mucosal (M side) of (**A**) the cecum and (**B**) the middle large intestine of Cldn15 KO mice. The box plots represent the minimum, first quartile, median, third quartile, and maximum values, the mean is marked with an X, n = 5 and 5–6, KO and WT, respectively. Statistical significance from left to right: (**A**) *P = 0.004; n.s. P = 0.177; n.s. P = 0.126; n.s. P = 0.931; (**B**) n.s. P = 0.548; n.s. P = 0.421; n.s. P = 0.690; n.s. P = 0.841, Mann–Whitney *U* test, Cldn15 KO vs. WT. Please see supplementary table 5 for data points.

Finally, we evaluated that the effect of claudin-15 deletion on SCFA-induced *I*_*sc*_ because SCFA are important for the intestinal luminal environment and in the development of proper barrier function^40–45^. To investigate SCFA-induced *I*_*sc*_, the intestinal samples were prepared and mounted in Ussing chambers under short-circuited conditions. SCFA (acetate, propionate, or butyrate, as Na^+^ salts) were added for a final concentration of 10 mM to the mucosal side chamber and the change of *I*_*sc*_ was measured (Fig. 10, Supplementary Table 5).

In the cecum of Cldn15 KO mice *I*_*sc*_ increment was larger for acetate (p = 0.004) and propionate (p = 0.126) (Fig. 10A). Upon addition of 10 mM propionate, the *I*_*sc*_ immediately increased and then decreased, revealing a sharp peak. However, since this change occurred immediately, it is not likely to be an effect of propionate transporter activity, but some other effect. Butyrate-induced *I*_*sc*_ increment did not change in the cecum of Cldn15 KO mice (Fig. 10A). No differences in SCFA-induced *I*_*sc*_ increment were observed in the middle large intestine of Cldn15 KO and WT mice (Fig. 10B).

## Discussion

The permselectivity and the role of the paracellular pathway in the cecum and large intestine have not been well defined, so the goal of this research was to investigate paracellular pathways in the cecum and large intestine, using claudin-15 knockout (Cldn15 KO) mice. We found that claudin-15 is responsible for ion selectivity and transepithelial conductance in the cecum and large intestine, and the loss of claudin-15 resulted in a decreased cationic selectivity in the paracellular pathway, which merits a discussion on the importance of Na^+^ in the luminal contents and the paracellular pathway in the cecum and large intestine.

The large intestine is considered to be an “organ of conservation” because of its absorptive ability^46^. Large intestinal epithelia are divided into two epithelia, surface epithelia and crypt epithelia. A long-held view was that surface epithelia are absorptive and crypt epithelia are secretory^47^; however, in recent years, crypts have been shown to be absorptive as well as secretory^48–50^. It is now understood that surface and crypt epithelia functions are not so strictly divided. The colonic epithelia are thought to salvage undigested energy sources via bacterial fermentation and the absorption of SCFA^51,52^, as well as absorption of Na^+^ and water and secretion of K^+^^48,53^.

Barrier function of epithelia is often quantified in terms of transmural resistance, which is thought to be mostly determined by the paracellular pathway where a leaky epithelia will have higher transcellular resistance than paracellular resistance, and a tight epithelia will have higher paracellular resistance relative to transcellular resistance^1,54,55^. The mouse distal large intestine has been analyzed using conductance scanning and transepithelial impedance analysis and it was determined to be a medium-tight epithelia. However, the surface epithelia was found to be tighter than the crypt epithelia^56^. The difference in leakiness of the surface and crypt epithelia suggests that paracellular transport has an important role in the crypts, and indeed the expression of claudin-2 and claudin-15 in the cecal and colonic epithelia supports this idea.

The results obtained in this study can be divided into 4 main points: (1) Tissue distribution of claudin-15 is related to paracellular function; (2) Similar to the small intestine, Na^+^ may be important for uptake of nutrients such as SCFA; (3) The paracellular pathway allows for energy efficiency similar to the kidney; and (4) Transcellular transport of Na^+^.

### Tissue distribution and function

In the small intestine, claudin-15 is expressed primarily in the villi and is thought to be play an important role in Na^+^-dependent absorption of nutrients. However, claudin-15 is strongly expressed in crypts in the cecum and large intestine and claudin-2 is expressed in the crypts at all sites in the small intestine, cecum, and proximal large intestine. Claudin-2 KO mice do not show decreased transepithelial conductance in the proximal large intestine^57^, suggesting the contribution to total transepithelial conductance from claudin-15 is higher than that of claudin-2. In the cecum, loss of claudin-15, which is expressed in the crypts, results in reduced transepithelial conductance and cation selectivity, despite the expression of claudin- 2. This indicates that claudin-15 plays a more important role in the epithelial function of the cecum. So, what is the function of claudin-15 in the cecum? One possibility is the presence of a Na^+^-dependent nutrient absorption mechanism in the cecum.

### SCFA absorption and nutrient absorption

While the small intestine is undisputedly the main site of nutrient absorption, it is thought that the cecum and large intestine can also absorb nutrients. Many nutrients, such as glucose, require the co-transport of Na^+^ for uptake in the small intestine^58,59^, and this is thought to be the reason that high luminal Na^+^ concentrations are maintained. Cldn15 KO mice have low luminal Na^+^ content in the small intestine^10,27^ as well as the cecum and large intestine. Originally, due to the low luminal Na^+^ concentration in Cldn15 KO mice, glucose uptake was thought to be decreased compared to WT mice^10^, leading to a high influx of undigested nutrients into the cecum. However, analysis of the cecal luminal contents from Cldn15 KO mice did not support this theory and metabolic cage experiments revealed that the RQ was not lower than WT mice, suggesting glucose uptake in the small intestine is not altered in Cldn15 KO mice.

It was found that intestinal transit time of the jejunum is slower in Cldn15 KO mice compared to WT mice, perhaps to allow for better nutrient absorption^8^. Glucose may be adequately absorbed; however, uptake of some nutrients may be impaired in the cecum due to the loss of claudin-15. The cecum and large intestine act as energy scavengers by absorbing the SCFA created by the fermentation of undigested nutrients by the intestinal bacteria^30,60–62^. SCFA absorption is thought to require cotransport of either Na^+^ or a proton via the SCFA transporters SMCT1 and monocarboxylate transporter (MCT) 1^11^. There were no great differences in SCFA-induced *I*_*sc*_ under high-Na^+^ conditions, except in the cecum, where the acetate-induced increment of *I*_*sc*_ was doubled, suggesting that acetate transport may be affected under physiological conditions in Cldn15 KO mice. In the large intestine, transport of SCFA was not greatly altered, suggesting that transport is not electrogenic or heavily dependent on Na^+^-coupled transport.

In addition to SCFA absorption, the large intestine may be responsible for amino acid absorption^63,64^. Ussing chamber analysis of the rat cecum and proximal large intestine found that amino acids are absorbed and, with exception of alanine, isoleucine, lysine, methionine, phenylalanine, and tyrosine, a significant component of Na^+^-dependent transport was observed^64^, revealing a possible requirement for luminal Na^+^ homeostasis, similar to the small intestine. A murine Na^+^-dependent amino acid transport system (B^0+^ amino acid transporter; mCATB^0+^) was found to induce Na^+^-dependent amino acid transport in mCATB^0+^-expressing *Xenopus* oocytes, and because mCATB^0+^ is strongly expressed in the murine large intestine^63^, it could be a candidate for Na^+^-coupled transport, mirroring that observed in the rat. The presence of claudin-15 in the cecum and large intestine may be necessary to support nutrient absorption and scavenging.

### Energy efficiency

In terms of transepithelial ion transport, tighter intercellular spaces are efficient, but the presence of ion permeability in the intercellular pathways is considered important for overall biological energy efficiency in tissues such as the kidney^6^. In WT mice, the transepithelial resistance is lower in the cecum and proximal large intestine and gradually increases along the intestinal axis. This suggests that the expression of barrier-like claudin proteins in the distal region is important for the formation of a large concentration gradient, which may be required for efficient absorption into the body from the lumen^1^. It has been theorized that large intestinal epithelium would have an anionic permselectivity, which would provide a flow of counter anions toward the lumen for electrogenic Na^+^ absorption in the distal large intestine^16^. However, our results confirm previously published results that the cecum and middle large intestine are cationic selective^18,19^, and we have identified claudin-15 is the molecule responsible for this cationic selectivity as it is lost after the deletion of claudin-15. Taken together with the decrease in transepithelial electrical conductance, it is possible that the localization of other TJ proteins is changed in Cldn15 KO mice. Claudin-7 appears to localize to the tricellular TJ in the cecum. Since claudin-7 is considered to be a barrier-forming claudin^39^, its presence in the TJ in the absence of claudin-15 would likely cause the TJ to become “tighter”, and indeed, the conductance and dilution potential decrease in the cecum of Cldn15 KO mice. The movement of claudin-7 to the TJ further supports the idea that claudin-15 has a significant role as a cation selective channel in the cecum. This phenomenon of claudin-switching has been observed in the kidney of claudin-10a KO mice^65^, where the loss of the anion selective claudin-10a results in a shift to cationic permeability via claudin-2 redistribution in the proximal tubule of those mice.

In Cldn15 KO mice, *I*_*sc*_ is much higher than the WT controls, suggesting that some transcellular mechanism is compensating for the loss of paracellular pathways. In claudin-2 KO mice, it was found that the loss of the paracellular Na^+^ pathway in the proximal tubule of the kidney is compensated for by transmembrane transport^6^. However this comes at the cost of energy efficiency, allowing the authors to make the conclusion that in the kidney, the paracellular pathway is an energy-conserving mechanism^6,7^. We propose that like claudin-2 in the kidney, claudin-15 expression in the paracellular pathway, while not indispensable under physiological conditions, as the main paracellular pathway for Na^+^ in the cecum and large intestine, it may play a role in energy efficiency by providing the Na^+^ required for nutrient uptake and scavenging and preventing loss of potential energy through lack of absorption of nutrients.

### Transcellular transport of Na^+^

Cecal Na^+^ flux was found to be decreased in both directions in Cldn15 KO mice but there was no difference in the middle large intestine. Addition of the NHE3 inhibitor S3226 resulted in decrease of both M→S flux in Cldn15 KO and WT mice, but *I*_*sc*_ was only profoundly affected in Cldn15 KO mice. This suggests that the higher *I*_*sc*_ in Cldn15 KO mice may be due to electrogenic processes such as Cl^−^ transport (for example via CFTR or basolateral NKCC1) or the action of a cation channel that may be affected by the inhibition of NHE3 or some other electrogenic process that is inhibited by S3226 via an unknown mechanism. A preliminary experiment with bumetanide (an NKCC1 inhibitor) revealed some inhibition of baseline *I*_*sc*_, suggesting CFTR may be activated in the cecum and middle large intestine of Cldn15 KO mice. However, more studies are required to understand the mechanisms behind the increased basal *I*_*sc*_ in Cldn15 KO mice.

In summary, the paracellular mechanisms and characteristics of the cecum and large intestine have not been well studied and as a result, important physiological details, including permselectivity and ionic homeostasis mechanisms remain unidentified. Our study shows for the first time that claudin-15 is the molecule responsible for ion selectivity and transepithelial conductance in the cecum and large intestine. Loss of claudin-15 alters the intestinal environment, and it also alters the function of the cecum and large intestine. Going forward, more studies are needed to understand the role of the paracellular pathway in the cecum and large intestine.

## Materials and methods

All authors have been given access to the study data and have reviewed and approved this manuscript.

### Animals

Cldn15 KO mice on a C57BL/6 J background were obtained the Laboratory Animal Resource Bank (National Institutes of Biomedical Innovation, Health and Nutrition, Japan). C57BL/6 J mice were purchased from Clea (Tokyo, Japan).

Mice were maintained in the animal care facility at the University of Shizuoka. Animal experiments were approved by the Animal Care and Use Committee of the University of Shizuoka (permit #205272 and #656-2303) and were conducted following the guidelines for animal experimentation by the University of Shizuoka and the ARRIVE guidelines. C57BL/6 J mice were used as controls. Mice aged 2–10 months old (please see Supplementary Table 7) were used in experiments. Mice were given water and fed a standard pellet diet (CE-2, Clea, Tokyo, Japan) ad libitum and housed in a temperature and humidity-controlled environment with a 12-h light/dark cycle.

### Anaesthesia and euthanasia

Mice were anaesthetized by either an intraperitoneal injection containing a mixture of three drugs (10 µL/g body weight) consisting of medetomidine (30 µg/mL; Nippon Zenyaku Kogyo, Fukushima, Japan), midazolam (0.4 mg/mL; Teva Pharma, Nagoya, Japan), and butorphanol (0.5 mg/mL; Meiji Seika, Tokyo, Japan), or by isoflurane (Wako Pure Chemical Industries, Osaka, Japan) at a flow rate of 1.5 L/min and 2–3% isoflurane as administered by an animal anesthetizer (TK-7; Bio Machinery, Funabashi, Japan). In all cases mice were checked for adequate anaesthesia by a negative pedal response and animals were quickly euthanized by cervical dislocation after the experiments.

### Metabolome analysis

Luminal contents (30–50 mg) from the cecum were collected and weighed and water-soluble metabolites were extracted as per the instructions provided Human Metabolome Technologies (HMT; Tsuruoka, Japan). CE-TOFMS analysis in anion and cation mode was performed on an Agilent CE-TOFMS system (Agilent Technologies Inc.) by HMT. The peak values obtained were normalized to cationic and anionic internal standards. The compounds were identified by comparison to the metabolites registered in the HMT metabolite library and where possible linked to other databases.

### Indirect calorimetry

Respiratory gas analysis was performed with an Arco-2000 (Arco System, Chiba, Japan) as described previously^66^. Mice were acclimatized for 2 days and measurements began on the third day. Fasting measurements began at 9 am and ended at 9 am on the next day. Refeeding measurements consisted of adding a similar size pellet of food into the cage and measuring for 3 h. After refeeding was done, the food was collected and weighed, and the amount of food eaten was calculated. Locomotor activity was measured using an automated motion analysis system.

### Luminal contents analysis

Luminal contents from the cecum, proximal and distal large intestine were collected as previously described^67^. Briefly, the intestinal tract was removed after application of anaesthesia. The cecum and large intestine were washed in 300 mM mannitol and each segment was opened and the luminal contents were collected in pre-weighed and pre-dried tubes. The tubes were then weighed again and set to dry in a drying oven at 80 °C for 24 h. After drying the tubes were measured again and the water percentage was calculated. Then set amounts of deionized water were added to the tubes and the dried luminal contents were heated for 2 h at 80 °C and mashed with a spoon to break down the solid material. After heating the dissolved luminal contents were centrifuged for 10 min at 12,000 RPM. The liquid phase was then collected and put into fresh tubes. Na^+^ and K^+^ concentrations were analyzed using electrodes (LAQUAtwin; Horiba, Kyoto, Japan). Cl^−^ concentration was assessed by a Cl^−^ electrode (Radiometer PHM250 Ion Analyzer, Villeurbanne, France).

### Flux, electrical conductance, and SCFA-induced *I*_*sc*_ measurements

The transepithelial flux and the measurement of transepithelial electrical conductance (*G*_*t*_) and short circuit current (*I*_*sc*_) under short-circuit conditions were performed as previously described with modifications^68^. Briefly, mice were anaesthetized with an injection containing a mixture of three drugs (same as above). The cecum and the large intestine were removed, and the large intestine was divided into three segments equal in length (proximal, middle, and distal). Each colon segment and the cecum were opened longitudinally. Intestinal sheets were rinsed in ice cold Ringer’s solution (containing in mM: 119 NaCl, 21 NaHCO_3_, 2.4 K_2_HPO_4_, 0.6 KH_2_PO_4_, 1.2 CaCl_2_, 1.2 MgCl_2_, 10 D-glucose, and 10 µM indomethacin) gassed with 95% O_2_-5% CO_2_ (pH 7.4). The muscle layer was removed by blunt dissection and the resulting intestinal preparations were then mounted in Ussing chambers (window area of 0.2 cm^2^, bathed in 5 mL Ringer’s solution kept at 37 °C). As described by Ishizuka et al.^8^, intestinal preparations were continuously short-circuited by a voltage-clamping amplifier (CEZ9100; Nihon Kohden, Tokyo, Japan). The *I*_*sc*_ was considered positive with current flowing from the mucosal side (M side) to the serosal side (S side). *G*_*t*_ was calculated according to Ohm’s Law, using the change in current after ± 10 mV pulses. The unidirectional transepithelial radio-active isotope flux of ^22^Na^+^ in both the mucosal-to-serosal (M→S) and the serosal-to-mucosal (S→M) direction was measured as described previously^8^. Briefly, at the start and end samples from the labeled side were taken, and during the experiment samples (0.5 mL each) were taken from the unlabeled “cold” side every 20 min and immediately replaced with an equal volume of unlabeled solution. Samples were counted in a liquid scintillation counter (LSC-7500; Aloka, Tokyo, Japan).

### Dilution potential measurement

To assess the paracellular pathway, preparations from the cecum and the large intestine were prepared as above. The intestinal preparations were mounted in Ussing chambers with a window diameter of 5 mm. As described previously^35^, the intestinal preparation was bathed in HEPES buffer (pH 7.4, 37 °C, gassed with 100% O_2_) containing in mM: 10 HEPES, 150 NaCl, 1 MgCl_2_, 2 CaCl_2_, 10 D-glucose, and 10 µM indomethacin. Electrical parameters were measured under open-circuit conditions and Ohm’s law was used to calculate transmural conductance and equivalent *I*_*sc*_. To measure the dilution potential, the buffer on one side (either M or S side) was replaced with the above HEPES buffer containing 75 mM NaCl and 150 mM mannitol. By diluting the NaCl concentration on either the mucosal (M) side or serosal (S) side of the intestinal membrane, the selectivity of the tight junctions can be investigated by looking at the change in the transepithelial potential difference as Na^+^ and Cl^−^ move through the tight junctions towards equilibrium. The relative permeabilities of Na^+^ and Cl^−^ can be calculated by Goldman-Hodgkin-Katz equation^34^ and the absolute permeabilities of the tight junctions can be estimated by the Kimizuka-Koketsu equation^36^. The change in transmucosal potential difference after dilution was measured and used to calculate the permeability of Na^+^ (P_Na_) and Cl^−^ (P_Cl_) as well as the charge selectivity of the paracellular pathway (P_Na_/P_Cl_).

### Immunofluorescence

The large intestine and the cecum were excised, and the large intestine was divided into 3 segments as in the Ussing chamber experiments. Each segment was opened and rinsed with ice cold PBS. Each tissue segment was then submerged and coated with Tissue-Tek O.C.T. compound (Sakura Finetek, Tokyo, Japan), and placed in a plastic mold containing O.C.T. compound. The samples were snap frozen and kept at − 80 °C until thin sectioning. Specimen blocks were sectioned into 5 µm slices using a Cryostat (CM3050 S; Leica Biosystems, Nussloch, Germany), put on coverslips, and dried under a fan for 30 min. Next the coverslips were incubated in 95% ethanol on ice for 30 min, bathed in acetone for one minute, followed by washing 3 times in PBS for 5 min each. The tissue was blocked for unspecific binding with 5% skim milk powder in 0.1% Triton X-100 in PBS (0.1% PBST) for 60 min. The coverslips were incubated with primary antibodies for either occludin (rat anti-mouse occludin monoclonal antibody raised against MOC37)^69^ and claudin-2 (#51-6100, ThermoFisher Scientific, Rockford, IL), claudin-7 (#34,9100, ThermoFisher Scientific, Rockford, IL) or claudin-15 (Kindly provided by Prof. Furuse, National Institute of Physiological Science, Okazaki, Japan)^70^ for 60 min. After washing in PBS, coverslips were incubated with secondary antibodies (1:1000 dilution) conjugated with Alexa Fluor 488 (Abcam, Cambridge, UK) or Alexa Fluor 546 (Invitrogen, Carlsbad, CA). After washing, the coverslips were mounted onto glass slides with mounting medium (Fluoromount-G; SBA Southern Biotechnology Associates, Inc., Birmingham, AL). Tissue was visualized using a laser scanning microscope (LSM700; Zeiss, Oberkochen, Germany).

### Statistical analyses

Values obtained in the experiments are given as the mean (standard deviation; SD) of the indicated number of animals. Results expressed as box plots show the minimum, first quartile, median, third quartile, and maximum values. Mann–Whitney *U* test was used for comparisons between two groups. Statistical analysis was performed by IBM SPSS Statistics software (Version 28.0.0.0 (190)). Statistical analysis for the metabolome data was performed by Human Metabolome Technologies (HMT; Tsuruoka, Japan). In all instances, P < 0.05 was considered to be statistically significant.

## Supplementary Information

Supplementary Tables.

## Data Availability

Data, analytic methods, and study materials are all available on request to the corresponding author.

## Abbreviations

CE-TOF: Capillary electrophoresis time-of-flight
ENaC: Epithelial sodium channel
*G*_*t*_: Transepithelial electrical conductance
*I*_*sc*_: Short-circuit current
KO: Knockout
M side: Mucosal side
M→S: Mucosal to serosal
MCT1: Monocarboxylate transporter 1
NHE3: Sodium-hydrogen exchanger 3
P_Cl_: Absolute permeability coefficient of Cl^−^
P_Na_: Absolute permeability coefficient of Na^+^
P_Na_/P_Cl_: Relative permeability of Na^+^ over Cl^−^
PBS: Phosphate-buffered saline
PCA: Principal component analysis
PD: Potential difference
RQ: Respiratory quotient
S side: Serosal side
S→M: Serosal to mucosal
SD: Standard deviation
SCFA: Short-chain fatty acids
SGLT-1: Sodium-glucose transporter 1
SMCT1: Sodium-coupled monocarboxylate transporter 1
TJ: Tight junction(s)
WT: Wild type
