# Supplementary material for: The effect of claudin-15 deletion on cationic selectivity and transport in paracellular pathways of the cecum and large intestine
Source: Sci Rep. 2023 Apr 26;13:6799. doi: 10.1038/s41598-023-33431-5 (PMC10133298; doi:10.1038/s41598-023-33431-5)
Supplement: Supplementary file 1 — Supplementary Tables. [file 41598_2023_33431_MOESM1_ESM.pdf]

The effect of claudin-15 deletion on cationic selectivity and transport of paracellular pathways in the cecum and large intestine

Wendy Hempstock[1,2], Nozomi Nagata[1], Noriko Ishizuka[1] and Hisayoshi Hayashi[1*]

[1] Laboratory of Physiology, Graduate School of Nutritional and Environmental Sciences, University of Shizuoka, 52-1 Yada, Suruga-ku, Shizuoka, 422-8526, Japan.

[2] Department of Nursing, School of Nursing, University of Shizuoka, 52-1 Yada, Suruga-ku, Shizuoka, 422-8526, Japan.

* hayashih@u-shizuoka-ken.ac.jp

Correspondence:

Hisayoshi Hayashi

Laboratory of Physiology, Graduate School of Nutritional and Environmental Sciences,

University of Shizuoka, 52-1 Yada, Suruga-ku, Shizuoka, Shizuoka, 422-8526, Japan.

Email: hayashih@u-shizuoka-ken.ac.jp

Tel/Fax: +81-054-264-5535

Wendy Hempstock whempstock@u-shizuoka-ken.ac.jp

Nozomi Nagata bidadaradara@icloud.com

Noriko Ishizuka n-ishizuka@u-shizuoka-ken.ac.jp

Running Title:

The role of claudin-15 in the cecum

Supplementary table 1: Detailed data for figure 4.

	Cecum		Prox LI		Distal LI	
	WT	KO	WT	KO	WT	KO
Sodium	103.1	22.4	88.5	34.2	57.5	33.9
	94.4	34.0	88.2	46.3	45.4	43.8
	105.8	22.5	117.3	48.0	87.7	50.4
	90.8	46.2	88.3	72.2	64.1	60.1
average	98.5	31.2	95.6	50.2	63.6	47.0
SD	7.1	11.3	14.5	15.9	17.8	11.0
N	4	4	4	4	4	4
**P**	**0.029**		**0.029**		**0.2**	
Potassium	75.4	173.1	55.4	90.9	80.0	192.3
	59.9	167.9	46.1	131.0	55.4	144.3
	81.1	150.1	65.6	140.0	85.0	176.4
	64.3	177.5	47.7	96.6	57.6	134.7
average	70.2	167.2	53.7	114.6	69.5	161.9
SD	9.8	12.0	8.9	24.5	15.2	27.0
N	4	4	4	4	4	4
**P**	**0.029**		**0.029**		**0.029**	
Chloride	52.6	79.3	70.5	87.2	23.2	75.2
	35.6	71.2	88.2	108.2	31.4	55.4
	21.9	72.3	47.4	112.8	26.5	76.8
	40.9	71.7	74.2	68.9	46.7	78.8
average	37.8	73.6	70.1	94.3	31.9	71.6
SD	12.8	3.8	16.9	20.2	10.4	10.9
N	4	4	4	4	4	4
**P**	**0.029**		**0.343**		**0.029**	
Water	78.6	84.1	75.1	78.7	58.7	60.7
	75.9	78.8	76.2	80.5	63.2	62.9
	74.5	81.7	72.1	81.6	59.9	58.9
	73.4	80.4	73.4	70.0	62.5	54.3
average	75.6	81.3	74.2	77.7	61.1	59.2
SD	2.2	2.2	1.8	5.3	2.1	3.6
N	4	4	4	4	4	4
**P**	**0.029**		**0.343**		**0.686**	

Supplementary table 2: Detailed data for figure 5.

	Cecum		Middle LI	
	WT	KO	WT	KO
Conductance	39.97	17.41	28.85	23.28
	44.68	13.38	26.11	23.16
	28.97	23.52	22.60	26.46
	25.42	17.48	15.85	24.12
	24.13	33.97	20.07	19.36
	22.56	10.00	16.82	26.01
	25.00	9.12		
average	30.1	17.8	21.7	23.7
SD	8.7	8.7	5.1	2.5
N	7	7	6	6
**P**	**0.017**		**0.485**	
Isc	3.53	2.59	2.25	2.35
	-0.09	2.61	1.23	1.19
	0.94	3.32	1.86	3.08
	0.30	4.29	1.13	2.49
	-0.14	4.13	1.06	2.74
	-0.28	1.81	1.35	2.35
average	0.7	3.1	1.5	2.4
SD	1.5	1.0	0.5	0.6
N	6	6	6	6
**P**	**0.026**		**0.026**	

Supplementary table 3: Detailed data for figure 6.

Cecum M-->S Flux						
WT	10	30	50	70	90	110
	37.38	37.22	35.95	25.83	29.45	20.67
	60.14	66.28	62.44	41.19	24.11	
	54.98	53.21	55.08	26.16	22.19	24.44
	28.93	16.64	66.11	21.34	9.68	20.16
Avg	45.3575	43.3375	54.895	28.63	21.3575	21.75667
SD	14.65854	21.40097	13.4369	8.657147	8.369028	2.337784
N	4	4	4	4	4	3
KO	10	30	50	70	90	110
	34.2	35.4	32.9	11.78	8.11	11.5
	21.1	21.78	20.38	17.95	13.61	15.69
	25.37	27.51	21.92	17.65	13.16	8.16
	26.62	27.58	30.4	29.68	19.93	16.83
	16.15	26.5	15.83	6.96	6.5	7.13
Avg	24.688	27.754	24.286	16.804	12.262	11.862
SD	6.71653	4.894188	7.140391	8.514184	5.288229	4.346328
N	5	5	5	5	5	5
**P (WT vs KO)**	**0.032**	**0.286**	**0.016**	**0.111**	**0.111**	**0.036**

Cecum S-->M Flux						
WT	10	30	50	70	90	110
	13.2	11.4	12.76	10.38	12.23	12.5
	18.19	22.7	22.78	8.74		
	16.49	16.98	16.65	18.05	12.5	23.2
	14.74	15.76	14.47	13.02	11.67	9.73
	10.58	12.23	12.14	11.6	10.42	11.47
	14.65	14.13	13.68	16.53	14.67	7.14
avg	14.64167	15.53333	15.41333	13.05333	12.298	12.808
sd	2.630296	4.085857	3.935641	3.603297	1.548635	6.153842
N	6	6	6	6	5	5
KO	10	30	50	70	90	110
	5.82	10.24	5.93	6.21	4.4	2.82
	12.31	11.04	9.24	5.45	4.81	4.34
	9.84	9.01	11.09	9.78	11.44	8.65
	1.94	12.33	8.04	4.73	7.03	5.35
	14.69	12.33	17.54	14.37	13.23	19.79
	3.2	2.01	2.27	1.36	1.66	0.94
Avg	7.966667	9.493333	9.018333	6.983333	7.095	6.981667
SD	5.123029	3.880127	5.157416	4.517361	4.439481	6.786732
N	6	6	6	6	6	6
**P**	**0.026**	**0.026**	**0.041**	**0.041**	**0.082**	**0.082**

Middle LI M-->S Flux						
WT	10	30	50	70	90	110
	64.66	59.44	69	18.21	34.39	24.53
	34.56	40.62	34.84	27.21	18.41	19.28
	52.39	53.34	63.92	73.39	16.95	
	35.36	38.63	33.04	23.37	10.54	12.28
	27.38	26.79	21.9	17.73	8.06	8.35
Avg	42.87	43.764	44.54	31.982	17.67	16.11
SD	15.25476	12.8627	20.69291	23.47524	10.29336	7.207214
N	5	5	5	5	5	4
KO	10	30	50	70	90	110
	9.36	11.58	14.36	13.21	12.54	
	38.16	39.48	38.74	26.81	24.77	19.73
	36.93	33.96	50.48		45.27	18.67
	39.04	39.44	42.6	23.44	24.32	14.58
	19.69	19	20.77	16.19	13.03	16.11
Avg	28.636	28.692	33.39	19.9125	23.986	17.2725
SD	13.41021	12.71227	15.22268	6.292834	13.27384	2.351827
N	5	5	5	4	5	4
**P (WT vs KO)**	**0.548**	**0.151**	**0.548**	**0.413**	**0.421**	**1**

Middle LI S-->M Flux						
WT	10	30	50	70	90	110
	15.8	15.76	16.9	14.92	13.58	15.72
	10.32	7.98	9.41	10.04	11.69	8.08
	10.78	11.05	11.55	9.55	8.42	6.49
	7.12	7.93	8.26	5.5	10.79	6.79
	8.82	8.95	7.69	10.33	9.22	11.03
	20.03	9.68	12.98	6.03	7.26	8.97
Avg	12.145	10.225	11.13167	9.395	10.16	9.513333
SD	4.838023	2.951154	3.461811	3.415674	2.31324	3.456291
N	6	6	6	6	6	6
KO	10	30	50	70	90	110
	9.32	7.18	9.44	9.09	8.83	12.04
	8.31	8.5	9.31	8.46	10.64	9.02
	11.16	8.91	7.34	10.34	7.7	9.71
	12.68	12.43	10.95	11.7	11.36	15.58
	8.37	11.02	7.68	6.25	7.04	4.57
	13.22	12.38	13.44	9.08	7.78	9.48
Avg	10.51	10.07	9.693333	9.153333	8.891667	10.06667
SD	2.159222	2.189557	2.254752	1.833747	1.74572	3.635138
N	6	6	6	6	6	6
**P**	**0.818**	**1**	**0.485**	**1**	**0.31**	**0.589**

Supplementary table 4: Detailed data for figure 7.

	Cecum		Middle LI	
	WT	KO	WT	KO
Dilution Potential	7.5	0.2	4.6	0.3
	9.4	2.3	5.7	1.1
	5.3	1.8	6.4	2.1
	2.3	1.3	4.6	1.2
	5.6	1.6	1.9	1.7
average	6.0	1.4	4.6	1.3
SD	2.7	0.8	1.7	0.7
N	5	5	5	5
**P**	**0.008**		**0.016**	
PNa/PCl	2.641	1.018	1.768	1.037
	3.574	1.323	2.046	1.136
	1.939	1.244	2.253	1.283
	1.321	1.170	1.764	1.156
	2.013	1.206	1.258	1.228
average	2.298	1.192	1.818	1.168
SD	0.9	0.1	0.4	0.1
N	5	5	5	5
**P**	**0.016**		**0.016**	
PNa	7.557E-05	1.777E-05	6.568E-05	4.572E-05
	6.737E-05	2.006E-05	5.965E-05	5.936E-05
	1.031E-04	1.861E-05	6.939E-05	5.291E-05
	1.234E-04	2.445E-05	5.921E-05	3.833E-05
	6.942E-05	2.479E-05	5.714E-05	3.905E-05
average	8.8E-05	2.1E-05	6.2E-05	4.7E-05
SD	2.5E-05	3.3E-06	5.1E-06	9.1E-06
N	5	5	5	5
**P**	**0.008**		**0.032**	
PCl	2.862E-05	1.745E-05	3.714E-05	4.409E-05
	1.885E-05	1.516E-05	2.915E-05	5.227E-05
	5.317E-05	1.496E-05	3.079E-05	4.124E-05
	9.341E-05	2.090E-05	3.356E-05	3.317E-05
	3.449E-05	2.056E-05	4.541E-05	3.180E-05
average	4.6E-05	1.8E-05	3.5E-05	4.1E-05
SD	2.9E-05	2.8E-06	6.5E-06	8.4E-06
N	5	5	5	5
**P**	**0.032**		**0.421**	

Supplementary table 5: Detailed data for figure 10.

	Cecum		Middle LI	
	WT	KO	WT	KO
Acetate	9.6	12.2	3.2	8.0
	8.2	10.2	-2.4	3.2
	3.1	15.3	3.2	11.9
	8.2	20.4	4	3.2
	10.2	19.4	19.1	4.8
	3.1			
average	7.1	15.5	5.4	6.2
SD	3.2	4.4	8.1	3.7
N	6	5	5	5
**P**	**0.004**		**0.548**	
Prop. Peak	105.1	175.3	3.2	9.6
	51.0	55.0	0.0	8.0
	81.5	126.4	4.8	54.1
	71.3	159.0	11.9	6.4
	66.2	126.4	12.7	9.6
	154.9			
average	88.3	128.4	6.5	17.5
SD	37.2	46.2	5.6	20.5
N	6	5	5	5
**P**	**0.177**		**0.421**	
Propionate	1.6	10.2	12.7	6.4
	6.1	6.1	6.4	3.2
	9.2	10.2	3.2	0.0
	4.1	8.2	4.0	3.2
	7.6	8.2	0.0	6.4
	8.2			
average	6.1	8.6	5.3	3.8
SD	2.85	1.71	4.75	2.68
N	6	5	5	5
**P**	**0.126**		**0.69**	
Butyrate	1.6	4.1	4.8	6.4
	-2.0	2.0	3.2	6.4
	4.1	2.0	3.2	1.6
	6.1	4.1	4.0	-6.4
	2.5	3.1	6.4	9.6
	7.1			
average	3.2	3.1	4.3	3.5
SD	3.3	1.1	1.3	6.2
N	6	5	5	5
**P**	**0.931**		**0.841**	

Supplementary table 6: Detailed data for table 3.

	Cecum		Middle LI	
	WT	KO	WT	KO
M-->S flux	36.85	34.17	64.37	11.77
	62.96	21.09	36.68	38.79
	54.42	24.93	56.55	40.46
	37.23	28.2	35.68	40.36
		19.49	25.36	19.82
average	47.9	25.6	43.7	30.2
SD	13.0	5.9	16.1	13.5
N	4	5	5	5
**P**	**0.016**		**0.548**	
S-->M flux	12.45	7.33	16.16	8.65
	21.22	10.87	9.24	8.71
	16.7	9.98	11.13	9.14
	14.99	7.44	7.77	12.02
	11.65	14.85	8.49	9.02
	14.16	2.49	14.23	13.01
average	15.2	8.8	11.2	10.1
SD	3.5	4.1	3.4	1.9
N	6	6	6	6
**P**	**0.015**		**0.818**	
M-->S Post S3226	25.32	10.47	25.71	12.875
	32.65	15.75	21.63	23.77
	24.27	12.99	58.22	12.13
	17.06	22.15	15.4	20.78
		6.87	11.38	15.11
average	24.8	13.6	26.5	16.9
SD	6.4	5.8	18.6	5.1
N	4	5	5	5
**P**	**0.032**		**0.421**	
S-->M Post S3226	11.7	4.48	14.74	9.99
	8.74	4.86	9.94	9.38
	17.96	9.95	8.15	9.25
	11.48	5.7	7.69	12.88
	11.16	15.8	10.19	5.95
	12.78	1.32	7.42	8.78
average	12.3	7.0	9.7	9.4
SD	3.1	5.1	2.7	2.2
N	6	6	6	6
**P**	**0.065**		**1**	
M-->S Net change due to S3226	11.53	23.7	38.66	-1.105
	30.31	5.34	15.05	15.02
	30.15	11.94	-1.67	28.33
	20.17	6.05	20.28	19.58
		12.62	13.98	4.71
average	23.0	11.9	17.3	13.3
SD	9.0	7.4	14.5	11.7
N	4	5	5	5
**P**	**0.19**		**0.841**	

Supplementary table 7: Age data for mice used in experiments.

Figure 2			
WT	Age (wk)	Age (d)	Age (Mo)
106-1	12	84	2 mo 23 d
106-2	12	84	2 mo 23 d
106-3	12	84	2 mo 23 d
KO	Age	Age (d)	Age (Mo)
149-154	17	123	4 mo 1 d
149-155	17	123	4 mo 1 d
149-156	17	123	4 mo 1 d

Figure 3			
WT	Age	Age (d)	Age (Mo)
141-139	35	252	8 mo 7 d
141-140	35	252	8 mo 7 d
141-136	39	280	9 mo 5 d
KO	Age	Age (d)	Age (Mo)
141-137	35	252	8 mo 7 d
148-152	20	143	4 mo 21 d
148-149	24	171	5 mo 18 d

Figure 4			
WT	Age (wk)	Age (d)	Age (Mo)
30-23	23	164	5 mo 11 d
30-26	31	217	7 mo 3 d
44-45	9	61	1 mo 30 d
48-56	22	153	5 mo
KO	Age (wk)	Age (d)	Age (Mo)
44-46	18	125	4 mo 2 d
47-52	17	119	3 mo 27 d
48-57	22	153	5 mo
50-61	15	105	3 mo 14 d

Figure 5	Cecum		
WT	Age (wk)	Age (d)	Age (Mo)
72-1	10	71	2 mo 10 d
72-2	11	78	2 mo 17 d
73-5	11	78	2 mo 17 d
74-1	13	90	2 mo 29 d
74-3	13	90	2 mo 29 d
136-118	17	125	4 mo 4 d
82-2	18	125	4 mo 3 d
KO	Age (wk)	Age (d)	Age (Mo)
7-1 F	18	125	4 mo 3 d
4-1	20	141	4 mo 19 d
4-2	21	148	4 mo 26 d
1-1	25	176	5 mo 24 d
136-117	17	125	4 mo 4 d
179-208	31	217	7 mo 3 d

Figure 6			
WT	Age (wk)	Age (d)	Age (Mo)
72-1	10	71	2 mo 10 d
72-2	11	78	2 mo 17 d
73-5	11	78	2 mo 17 d
74-1	13	90	2 mo 29 d
74-3	13	90	2 mo 29 d
136-118	17	125	4 mo 4 d
KO	Age (wk)	Age (d)	Age (Mo)
7-1 F	18	125	4 mo 3 d
4-1	20	141	4 mo 19 d
4-2	21	148	4 mo 26 d
1-1	25	176	5 mo 24 d
136-117	17	125	4 mo 4 d
179-208	31	217	7 mo 3 d

Figure 7			
WT	Age (wk)	Age (d)	Age (Mo)
77-1	24	169	5 mo 16 d
77-2	25	175	5 mo 22 d
77-4	26	183	5 mo 30 d
108-5	28	196	6 mo 12 d
108-6	28	197	6 mo 13 d
KO	Age (wk)	Age (d)	Age (Mo)
2-3	43	303	9 mo 28 d
2-4	44	310	10 mo 5 d
2-5	46	324	10 mo 19 d
171-176	21	152	5 mo 1 d
173-184	21	151	5 mo 0 d

Figure 10			
WT	Age (wk)	Age (d)	Age (Mo)
76-6	22	159	5 mo 6 d
77-1	24	169	5 mo 16 d
77-2	25	175	5 mo 22 d
77-4	26	183	5 mo 30 d
108-5	28	196	6 mo 12 d
108-6	28	197	6 mo 13 d
KO	Age (wk)	Age (d)	Age (Mo)
2-3	43	303	9 mo 28 d
2-4	44	310	10 mo 5 d
2-5	46	324	10 mo 19 d
171-176	21	152	5 mo 1 d
173-184	21	151	5 mo 0 d
